# The Problems of Radiofrequency Ablation as an Approach for Advanced Unresectable Ductal Pancreatic Carcinoma

**DOI:** 10.3390/cancers2031419

**Published:** 2010-07-01

**Authors:** Raffaele Pezzilli, Claudio Ricci, Carla Serra, Riccardo Casadei, Francesco Monari, Marielda D’Ambra, Roberto Corinaldesi, Francesco Minni

**Affiliations:** 1 Department of Internal Medicine and Gastroenterology, S. Orsola-Malpighi Hospital, University of Bologna, Bologna, Italy; E-Mail: carla.serra@aosp.bo.it (C.S.); roberto.corinaldesi@unibo.it (R.C.); 2 Department of Surgery, S. Orsola-Malpighi Hospital, University of Bologna, Bologna, Italy; E-Mails: claudiochir@gmail.com (C.R.); riccardo.casadei@aosp.bo.it (R.C.); fraseo@libero.it (F.M.); marielda.dambra@hotmail.it (M.D’A.); francesco.minni@unibo.it (F.M.)

**Keywords:** pancreatic neoplasms, catheter ablation, surgery

## Abstract

Advanced ductal pancreatic carcinoma (PC) remains a challenge for current surgical and medical approaches. It has recently been claimed that radiofrequency ablation (RFA) may be beneficial for patients with locally advanced or metastatic PC. Using the MEDLINE database, we found seven studies involving 106 patients in which PC was treated using RFA. The PC was mainly located in the pancreatic head (66.9%) with a median size of 4.6 cm. RFA was carried out in 85 patients (80.1%) with locally advanced PC and in 21 (19.9%) with metastatic disease. Palliative surgical procedures were carried out in 41.5% of the patients. The average temperature used was 90 °C (with a temperature range of 30–105 °C) and the ratio between the number of passes of the probe and the size of the tumor in centimeters was 0.5 (range of 0.36–1). The median postoperative morbidity and mortality were 28.3% and 7.5%, respectively; the median survival was 6.5 months (range of 1–33 months). In conclusion, RFA is a feasible technique: however, its safety and long-term results are disappointing; Thus, the RFA procedure should not be recommended in clinical practice for a PC patient.

## 1. Introduction

Surgical resection is generally accepted as having a beneficial effect on survival in patients with adenocarcinoma [1]. Unfortunately, this is possible in only 5–25% of cases, with a five-year survival rate that does not exceed 29% in high volume centers [2] At the time of diagnosis, most patients have unresectable locally advanced disease with massive encasement of the major vessels (portal and superior mesenteric vein, superior mesenteric artery and/or hepatic artery) [3]. Vascular reconstruction in these patients can be carried out with acceptably low rate of morbidity and mortality without significant survival benefit [4]. Chemotherapy generally confers symptomatic improvement in these patients, improves the quality of life and prolongs survival; chemoradiation does not appear to be superior to chemotherapy [5].

**Figure 1:**
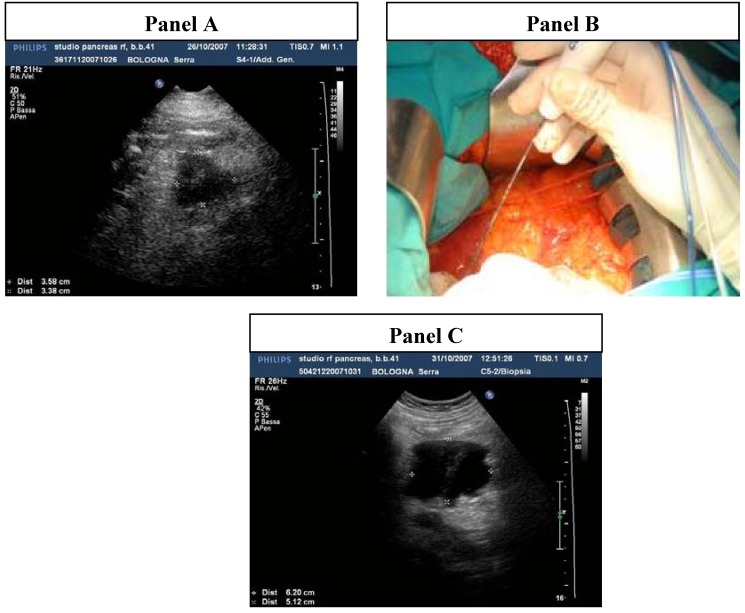
Radiofrequency ablation (RFA) and ultrasonographic (US) evaluation of the technique in a patient with locally advanced pancreatic head carcinoma. **Panel A*.*** Intraoperative US showing the pancreatic adenocarcinoma; **Panel B*.*** Intraoperative RFA; **Panel C**. Intraoperative US showing the ablated carcinoma.

Radiofrequency ablation (RFA) is a local ablative method used for the palliative treatment of solid tumors [6,7,8,9,10,11,12] (Figure 1). RFA should be an attractive approach in patients with unresectable, locally advanced and non-metastatic pancreatic cancer. We have recently revised the literature in order to evaluate the clinical benefit of RFA in pancreatic adenocarcinoma [13] and in this review we report in detail our consideration on this technique.

## 2. Search Strategy

A search was made on February 26, 2010 using the MEDLINE/PubMed database (U.S. National Library of Medicine National Institutes of Health) in order to select the data existing in the literature on ductal pancreatic adenocarcinoma treated with RFA. The Medical Subject Headings (MESH) terms used were “pancreatic neoplasms” (explanatory variable) and “catheter ablation” (outcome variable). We identified additional studies through hand searches of bibliographies from primary studies, review articles and key journals; the search yielded 49 hits [14,15,16,17,18,19,20,21,22,23,24,25,26,27,28,29,30,31,32,33,34,35,36,37,38,39,40,41,42,43,44,45,46,47,48,49,50,51,52,53,54,55,56,57,58,59,60,61,62]. Only full text papers in the English language were considered; letters, reviews without original data and animal studies, as well as studies treating cancers other than pancreatic adenocarcinoma, were excluded (40 papers in total) [14,15,16,17,18,19,21,22,24,25,26,27,28,29,31,32,33,34,35,37,40,43,44,45,46,47,48,49,50,51,52,53,54,55,56,57,58,59,60,61]. Of the remaining nine papers, we also excluded four papers because they contained data reported previously [20,23,38,42]. Two studies [63,64] were found in the references of the primary studies and they were added to the study list. Thus, seven papers were considered useful for this systematic review [30,36,39,41,62,63,64].

## 3. Data Extraction

From each of the seven articles selected the following data were extracted and analyzed: number of patients undergoing RFA for pancreatic ductal adenocarcinoma, demographic data (sex and age), site of lesions (head or body-tail), size of lesions, extension of the disease, operative approach (surgical or radiological), surgical data (palliative surgery, type of palliative surgery, ratio between the number of the passes of probe and the size of lesions, rate of postoperative morbidity, re-laparotomy, mortality and crude survival). Discrepancies in the interpretation of the data were discussed by all the authors in order to reach a consensus.

Data are presented as frequency, median and range; follow-up data are also presented as crude survivals after surgery.

## 4. Results

The seven manuscripts [30,36,39,41,62,63,64] reported the data regarding a total of 106 patients on whom RFA was performed for ductal pancreatic adenocarcinoma; the characteristics of the 106 patients are summarized in Table 1. The median number of procedures reported per publication was 12 (range 1–50). The median age of patients was 62 years with a range of 27–85 years; the male:female ratio in the pooled data was 1.3:1. The site of the tumors was most frequently the head of the pancreas (71/106; 66.9%); the median tumor size was 4.6 cm (range 3–12). RFA was carried out on 85 patients (80.1%) with locally advanced pancreatic neoplasms for vascular encasement of the peripancreatic vessels and in 21 patients (19.9%) with metastatic pancreatic disease. Information on the approach was available in all studies.

The characteristics of the 106 procedures are summarized in Table 2.

**Table 1:** Demographics and clinical data of 106 patients who underwent radiofrequency ablation for pancreatic ductal adenocarcinoma. Data are reported as absolute number, frequency, median and range. NR = not reported.

Reference	No. of patients	Gender		Age (year)	Site of tumors		Tumor size (cm)	Extension of disease	
		**FemalesNo. (%)**	**MalesNo. (%)**	**Median (range)**	**HeadNo. (%)**	**Body-tail No. (%)**	**Median (range)**	**Locally advancedNo. (%)**	**MetastasesNo. (%)**
**Matsui [63]**	20	8 (40)	12 (60)	59 (45–77)	11 (55)	9 (45)	5.3 (3–10)	9 (45)	11 (55)
**Date [30]**	1	–	1	58	1	–	3	–	1
**Varshney [36]**	3	2 (66.7)	1 (33.3)	60 (48–66)	3 (100)	–	6.5 (5–8)	3 (100)	–
**Hadjicostas [64]**	4	2 (50)	2 (50)	70 (59–79)	3 (75)	1 (25)	8.5 (3–12)	4 (100)	–
**Wu [39]**	16	6 (37.5)	10 (62.5)	67 (42–89)	8 (50)	8 (50)	NR	11 (68.7)	5 (31.3)
**Spiliotis [41]**	12	6 (50)	6(50)	66.5 (61–79)	11 (91.7)	1 (8.3)	3.7 (3–10)	8 (66.7)	4 (33.3)
**Girelli [62]**	50	23 (46)	27 (54)	64.5 (54.5–74)	34 (68)	16 (32)	4 (3–5)	50 (100)	–
**Overall**	106	47 (44.3)	59 (56.7)	64.5 (58–70)	71 (66.9)	35 (33.1)	4.6 (3–12)	85 (80.1)	21 (19.9)

**Table 2:** Technical characteristics of radiofrequency ablation in the 106 patients. Data are reported as absolute number, frequency, median and range. DB: double bypass surgery (common bile duct-jejunostomy plus a gastrojejunostomy); CJ: cholecystojejunostomy; GB: gastric bypass; BB: biliary bypass (common bile duct-jejunostomy); PJ: pancreaticojejunostomy. * the number of gastrojejunostomies not reported; ** the authors reported one minute at 90 °C. NR = not reported.

Reference	No. of patients	Approach		Type of associated procedures	No. of passes/size	RFA parameters	
		**Surgical No. (%)**	**Radiological No. (%)**	**No. (%)**	**Ratio**	**Temperature °C median and (range)**	**Time (min) median and (range)**
**Matsui [63]**	20	20 (100)	–	–	NR	50	15
**Date [30]**	1	1 (100)	–	1 DB (100)	1	90	10
**Varshney [36]**	3	2 (66.7)	1 (33.3)	–	NR	NR	NR
**Hadjicostas [64]**	4	4 (100)	–	4 CJ * (100)	0.36	NR	NR
**Wu [39]**	16	16 (100)	–	–	NR	30 **	12 **
**Spiliotis [41]**	12	12 (100)	–	12 DB (100)	0.5	90	6.5 (2–7)
**Girelli [62]**	50	50 (100)	–	19 DB; 8 GB; 3 BB; 1 PJ (62)	NR	97.5 (90–105)	NR
**Overall**	106	105 (95.2)	1 (4.8)	48 (45.3)	0.5 (0.36–1)	90 (30–105)	11 (2–15)

Almost all the procedures were performed during laparotomy (105/106; 95.2%); only one thermoablation was CT-guided for a pancreatic neoplasm located in the body of the pancreas (4.8%). Other palliative surgical procedures were carried out in only 44 patients: 32 double bypasses (common bile duct-jejunostomy plus gastrojejunostomy, 66.7%), eight gastric bypasses (16.7%), four cholecystojejunostomies (8.3%), three common bile duct-jejunostomies (6.2%) and one pancreaticojejunostomy (2.1%). The ratio between the number of passes of the probe and the size of the tumor was calculated in only 17 procedures due to the lack of data and was 0.5 (0.36–1). Data regarding the thermal kinetic characteristics were available in five studies covering 99 patients. The median temperature used was 90 °C (range 30–105). The median time of duration for each application of the probe in the 49 patients providing this information was 11 minutes (range 2–5 min). 

Data about the postoperative course and long-term survival are summarized in Table 3. 

**Table 3:** Frequency of postoperative and long-term results for 106 patients who received radiofrequency ablation for pancreatic adenocarcinoma. Data are reported as absolute number, frequency, median and range. NR: Not reported.

Reference	No. of patients	Morbidity No. of cases (%)	Reoperation No. of cases (%)	Mortality No. of cases (%)	Crude Survival in months Median and (range)
**Matsui [63]**	20	3 (15)	1 (5)	3 (15)	3
**Date [30]**	1	1 (100)	–	–	3
**Varshney [36]**	3	2 (66.7)	–	–	9 (1–11)
**Hadjicostas [64]**	4	–	–	–	6.5 (3–12
**Wu [39]**	16	7 (43.7)	–	4 (25)	NR
**Spiliotis [41]**	12	4 (33.3)	–	–	33 (6–39)
**Girelli [62]**	50	13 (26)	3 (6)	1 (2)	NR
**Overall**	106	30 (28.3)	4 (3.7)	7 (7.5)	6.5 (1–33)

Regarding postoperative outcome, data were available in all the studies covering the 106 patients. The median postoperative morbidity was 28.3% (Table 4) and the mortality rate was 7.5. The rate of re-laparotomy was 3.7%. Median survival in the 40 patients providing this information was 6.5 months (range 1–33 months).

**Table 4:** Type and frequency of postoperative complications in 30 patients who received radiofrequency ablation for pancreatic adenocarcinoma. One or more complication may be present in the same patient.

Complication	No. of complication	(%)
Gastro-intestinal hemorrhage	8	22.9
Pancreatic fistula	5	14.3
Biliary leak	5	14.3
Portal vein thrombosis	4	11.4
Pseudocyst	3	8.6
Sepsis	2	5.7
Polyuria	1	2.9
Ascites	1	2.9
Pmeumonia	1	2.9
Liver failure	1	2.9
Anastomotic ulcer	1	2.9
Severe acute pancreatitis	1	2.9
Renal failure	1	2.9
Delayed gastric emptying	1	2.9
**Overall**	35	100.0

## 5. Discussion

RFA for locally advanced ductal adenocarcinoma of the pancreas appears to be an attractive proposition and many hepatobiliary surgical units currently have the equipment and technical expertise for ablation of unresectable liver tumors [65,66]. The concept of thermal ablation for unresectable tumors is increasingly accepted. However, there are critical differences between the role of RFA in unresectable lesions of the liver and a parallel application in pancreatic cancer. As suggested by Giovannini [67], the differences are mainly anatomical and biological. First of all, liver tumors are surrounded by areas of normal hepatic parenchyma, and extension of the zone of ablation beyond the tumor will not usually have adverse consequences, whereas the pancreatic lesions may be traversed by the distal common bile duct and be closely related to the duodenum, stomach, transverse colon and portal vein; thus, the risks of inadvertent thermal injury are considerable and become even more dangerous in the presence of an unresectable tumor. Secondly, hepatic metastases are usually discrete lesions, whereas locally advanced pancreatic cancer encases the vessels and extends retroperitoneally or proximally, rendering direct ablation of the entire tumor bulk impractical. An additional problem of this approach is the efficacy of thermal ablation of the pancreatic parenchyma, even if experimental studies have suggested an optimal temperature which should be used in a human setting [38]. In this systematic review, all these aspects were taken into consideration. In addition, we should point out that all the studies examined were retrospective and there were no randomized studies [30,36,39,41,62,63,64]. Furthermore, the analyses of the pooled data are critically influenced by the nature of their constituent reports. In the present study, bias is likely to have been introduced by variation in the selection of patients to undergo RFA, variation in surgical skill (some studies have safety and feasibility as a primary end point, others have survival), variation in disease staging, lack of standardization of RFA parameters and operative technique. 

The number of patients undergoing this type of surgery in any given series is small and the largest series [62] reported 50 consecutive patients. However, this latter study had two main biases: the first bias was that it was a retrospective study having a non-homogeneous population. In fact, 34 patients were initially considered for RFA; eight patients were treated with RFA after progression of known unresectable disease (three had previously been treated with chemotherapy alone and 5 had chemo-radiotherapy). Finally, eight patients were enrolled after progression of the disease during neoadjuvant therapy (two after chemotherapy alone and six after chemo-radiotherapy). The second bias of this study was that there was no standardized technique for the palliative surgery combined with radiofrequency ablation. 

All studies [30,36,39,41,62,63,64] confirmed that RFA was carried out for large lesions (median 4.6 cm), more frequently located in the head of the pancreas (nearly 70% of cases), in patients affected by unresectable pancreatic adenocarcinoma for locally advanced disease (80% of cases). The RFA was frequently performed using a surgical approach and only one case was carried out using a radiological approach alone [36]. This point is of paramount importance because the thermal injury of the peripancreatic vessels should be taken into account as a complication of the ablation of lesions localized in the pancreatic head as shown by Date *et al.* in a porcine model [38]. Furthermore, RFA may cause inadvertent thermal necrosis of the intra-pancreatic common bile duct or duodenum [34] and for this reason some authors [30,41,64] routinely performed a double surgical bypass in the lesions of the head. In contrast, in other studies, the palliative surgery was performed only when necessary [62] or was not carried out at all [36,39,63]. 

All studies published until now have demonstrated that RFA is a feasible technique in human pancreatic cancer, without intra-operative mortality, but only some studies have reported complete necrosis of the lesions, confirmed by intraoperative ultrasonographic examination. Another important aspect of the RFA procedure is the ratio of the number of passes of the probe and the size of the lesions; in only three studies this value was reported [30,41,64] and it was extremely variable, having a median of 0.5 with a large range (0.36–19); one possible explanation for this variability is due to the different devices used. 

The second critical point of this technique was the optimal thermal kinetic parameters (temperature and time). A reproducible temperature ablation was demonstrated by Date in 2005 in a porcine model [38]. Optimal thermal kinetic characteristics were produced by a target temperature of 90 °C applied for five minutes. At this temperature, there was ablation of the pancreatic tissue without injury to the adjacent viscera. Higher temperatures resulted in injury to the bile duct and the portal vein. In the studies analyzed, the temperature used for the thermal ablation with radiofrequency had a median value of 97.5 °C (range 30–105 °C) and the time of exposure had a median value of 11 minutes with a range of 2–15 minutes. 

Assessment of the morbidity from the pooled data was difficult to calculate from the reported data even if an overall postoperative morbidity rate of 28.3% seems to be realistic. The most frequent complication was the gastro-intestinal hemorrhage, followed by pancreatic fistula, biliary leak, portal vein thrombosis, pseudocyst and sepsis. The assessment of mortality was a more readily definable endpoint and was about 4% even if the endpoints of the studies were different because some studies have the outcome safety, others survival. 

Some authors [23,34] have suggested a relationship between the rate of complications and the temperature used. This correlation was unclear in this cohort of patients. In fact, Girelli *et al.* [62] reported a decrease of the overall complication rate from 24 to 8% when reducing the temperature from 105 °C to 90 °C. Wu *et al.* [39] also reported a high rate of postoperative morbidity using a temperature of 30 °C. The major postoperative complications appeared to be related to the RFA (septic shock, massive bleeding, portal thrombosis and pancreatic fistulas) [39,62] and to palliative surgery when a single or double bypass was carried out (most frequently a biliary leak) [41,62]. In conclusion, the RFA treatment is not a standardized approach; in fact, treatment parameters differ (*i.e.*, duration of ablation, temperature, approach radiological/surgical, combined surgical procedures) and in some studies thermal kinetic parameters were not given at all; thus, no stratification of the result can be performed.

The median crude survival was 6.5 months with a range of 1–33 months. Of importance, only two studies had overall survival as a primary end point. The first study [63] evaluated 20 patients with unresectable and metastatic carcinomas of the pancreas (all stage IV patients) and no statistical differences were found in the prognosis of these patients as compared to patients of the same stage who did not receive treatment with RFA. The second study [41] was carried out on 12 consecutive patients who underwent palliative therapy for unresectable pancreatic cancer plus RFA. The patients who were treated with palliative therapy plus RFA had a mean survival of 33 months. The survival rate was statistically significantly longer than survival in patients who underwent palliative surgery for unresectable pancreatic cancer. When the difference in survival rate was calculated for patients of the same stage, RFA provided a survival benefit, especially for patients with stage III pancreatic cancer, whereas stage IV patients had similar survival rates as compared to patients of the same stage who did not receive treatment with RFA.

Data regarding long-term pain control and quality of life assessment were not available in all the studies selected and this represented another drawback regarding the evaluation. Since inhomogeneous data of the reviewed papers reporting long-term pain control and quality of life assessment was a weak point of this analysis, we have recently carried out a prospective study analyzing the effects of RFA in patients with locally advanced/metastatic pancreatic adenocarcinoma [68]. The results were disappointing: of the seven patients enrolled, only four patients (57.1%) were eligible for treatment consisting of RFA that was carried out on the mobilized pancreatic head followed by biliary by-pass and gastro-jejunal-anastomosis. The RFA procedure was carried out in three of the four patients; in one case it was not performed due to upstaging of the neoplasia. The RFA achieved complete necrosis of the lesions in all three cases in which it was carried out, but a biliary fistula developed seven days after the procedure in one patient (33.3%), and all three patients developed ascites after a mean time of 8.6 days (range 7–9 days) from RFA. All patients died, at three, four and five months, respectively, after treatment. Thus, the study was interrupted by the high percentage of complications and for the early mortality.

## 6. Conclusions

RFA is a feasible procedure; however in contrast to that previously reported [69], its safety still remains under debate. The complication rate appears high without a clear benefit in terms of survival. Finally, based on the data of the literature, the RFA procedure is not recommended as palliative therapy in clinical practice for patients with unresectable pancreatic adenocarcinoma.
